# Urogenital myiasis in a post-menopausal rural woman: A case report

**DOI:** 10.1016/j.ijscr.2023.108138

**Published:** 2023-04-07

**Authors:** Abhigan Babu Shrestha, S.M. Samiul Hoque, Muhammad Hassnain Nawaz, Pashupati Pokharel, Sajina Shrestha, Abhishek Mahaseth

**Affiliations:** a M Abdur Rahim Medical College, Dinajpur, Bangladesh; b Maharajgunj Medical Campus, Institute of Medicine, Tribhuvan University, Kathmandu, Nepal; c KIST Medical College, Imadol, Patan, Nepal

**Keywords:** Urogenital myiasis, Maggots, Gynecology, Post-menopausal, Rural, Genital hygiene

## Abstract

**Background:**

Genital myiasis in females is a parasitic infection of the vulval region with the larva of various files species. Only a few cases of urogenital myiasis have been reported in the literature.

**Case presentation:**

We present a case of 55 years postmenopausal, farmer female otherwise healthy presenting to the outpatient department with complaints of maggots and severe itching in the vulval region. Examination revealed erythema in the labia major and groin without lymphadenopathy. In the vaginal examination; inflammation and a large number of maggots were observed in the urethral meatus, labia minora, and vaginal canal, progressing to the cervix. With this, she was diagnosed to be a case of urogenital myiasis. She was managed with the extraction of maggots using turpentine oil, along with broad-spectrum antibiotics and Foleys catheterization for a week. Later during follow up, she was asymptomatic and examination revealed no maggots, and the lesions were healed.

**Clinical discussion:**

Extraction of maggots along with symptomatic management is the mainstay of treatment of myiasis. A significant number of the adult population in the rural areas of developing countries are illiterate and are not familiarized with education regarding genital hygiene. So, along with physicians, policy makers should also be involved in public awareness for genital hygiene.

**Conclusion:**

Despite being rare, urogenital myiasis is preventable and treatable condition. Efforts at increasing genital hygiene awareness in a low resource country are utmost for its prevention.

## Highlights

• Urogenital myiasis is rare case with maggot infestation of the vulval region.
• Manual extraction of maggots using turpentine oil, along with symptomatic treatment is the mainstay of treatment.
• Public health awareness regarding maintenance of genital hygiene among the women of rural areas must be encouraged.

## 1 Introduction

Myiasis is a parasitic infection of living or dead tissues of a host by the larva of various species of flies. Entry of the larvae occurs through skin wounds or body cavities, such as the mouth, ears, eyes and urogenital tract. This kind of infestation in humans is more spread in humid and warm geographical conditions [1].

Genital myiasis is a rare condition with a handful of cases reported to date. The disease is associated with socio-economically poor regions, certain cultural habits, and favorable weather conditions for flies [2]. Involvement of the external parts of the female genitalia, especially the labia majora and minora, is often accompanied by symptoms such as tenderness, erythema and inflammation [3]. We report a case of urogenital myiasis in a post-menopausal woman from Bangladesh. This case report has been written in accordance to the SCARE criteria [4] and previous case report [5].

## 2 Presentation of case

A 55-year-old rural woman presented with the complaints of maggots in the vulval region along with severe genital itching. Her family's occupation was farming and animal (sheep, goats, and cows) husbandry, and belonged from a low economic status. She didn't have any other comorbidities like diabetes, and hypertension. She had a history of vulval ulcer and reported foul-smelling vaginal discharge for the past six months but had not visited the clinic due to fear of social stigma. She reported no history of underlying diseases, trauma, or insect bite to the vaginal area. Neither did she had a history of smoking cigarettes or hookah. In the past twelve months, the patient did not have sexual intercourse as her husband died 1 year ago. She is nulliparous and is in menopause. She reported wearing unclean underpants and skirts.

**Fig. 1:**
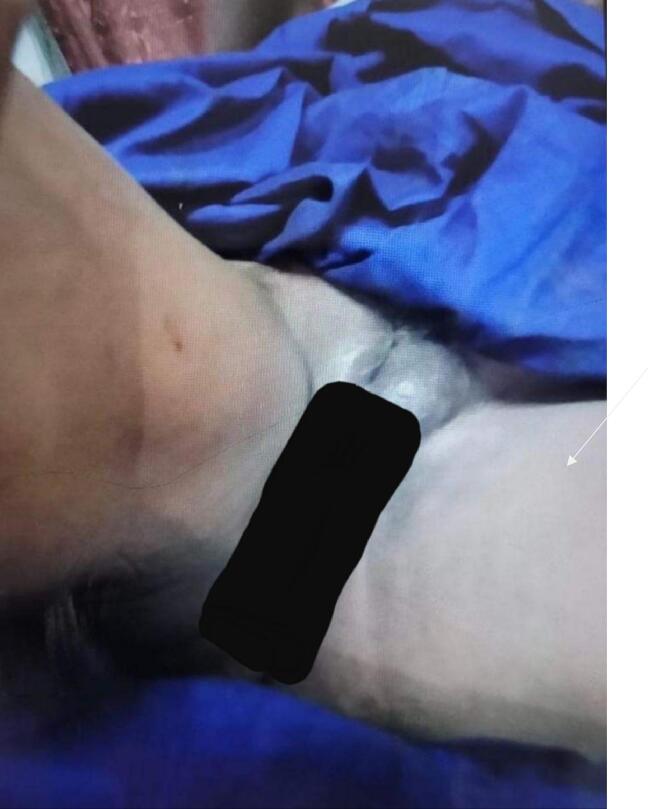
Elevated left side vulval area of the female with maggots.

The physical exam revealed erythema in the labia major and around the groins due to severe itching. In the vaginal examination, inflammation and a large number of maggots were observed in the urethral meatus, labia minora, and vaginal canal, progressing towards the cervix. Still, the cervix seemed intact on colposcopy. Infection and necrosis were observed in the labia minora and vaginal canal due to the existence of larvae that ate away the healthy tissues. Fig. 1 shows elevated left sided vulval area of the female with maggots.

She was mildly anemic. No significant lymphadenopathy was observed. Her blood pressure was 110/60 mm Hg and her pulse rate was 78 bpm. Per abdominal examination revealed a soft and tender lower abdomen.

The patient was managed initially by irrigation with turpentine oil by placing a Foley catheter in the vaginal tract. With intentions to kill the existing maggots and force the ones that might may lie deep in the tract to come out. Side by side broad spectrum antibiotics ceftriaxone (1 g daily) and clavulanic acid (1.2 g twice a day) were started and continued for 7 days. After irrigation with turpentine oil the necrotic tissue of labia minora and majora was excised and existing maggots were manually removed. A catheter was kept in situ for 7 days. She was advised to keep good hygiene and avoid wearing skirts while handling animals and their waste products. The patient returned to her normal routines and on subsequent follow up examinations, there was no larva present.

## 3 Discussion

Myia is Greek for fly and myiasis is an infestation of the skin by the developing larvae. Multiple species of fly are indigenous to various parts of the world. *Dermatobia hominis* (human botfly) is found in Central, and Southern America and parts of Mexico. *Cordylobia anthropophaga* (tumbu fly) is endemic in the sub-Saharan region and is attracted to animal faces and urine. *Cochliomyia hominivorax*/*Phaenicia sericata* lays their eggs near poorly managed wounds and larva feed on the dead necrotic tissue causing wound infection. *Hypoderma bovis*/*Gasterophilus intestinalis* infections usually occur in rural areas where animals are domesticated, but in humans, they are unable to complete their cycle so the infection is self-limiting in one to two weeks [3], [6], [7].

Myiasis started to be reported in medieval times and is usually restricted to warm environments like tropics and subtropical regions, but following the advent of frequent international travels individuals from any part of the world can be affected [8]. Larval infestation is known to be affecting the wounds, body orifices like the ear nose and umbilicus, but urogenital myiasis constitutes only 0.7 % of the total body larval infestation [9].

Prevalence of urogenital myiasis is predominantly more in the rural population, associated with poor self-cleanliness hygiene and nourishment. However, a systematic review showed no significant difference in myiasis cases in rural and urban areas [10]. Individuals with psychiatric disorders and genital prolapse have been reported to be more affected by it [11]. Baidya et al. reported a similar case with advanced vaginal malignancy [12]. Myiasis's predisposing factors include poor hygiene, poor socioeconomic, old age, lack of mobility, and ulcerated and prolapsed organs are predominantly involved [3], [6], [8], [9], [11]. In our case, the patient gave a history of being in close proximity and handling domestic animals and their waste products.

The commonly accepted mechanism for genital myiasis is that it occurs through fly eggs transferring through dusty clothes to the genitalia [13]. It has been shown previously that some villagers dry-wash their clothes on the ground, and flies lay eggs in the material leading to larvae attacking the host [14]. In addition to this, flies that lay eggs in genital cavities may have been attracted by the scent brought about by poor cleanliness and concurrent genital infections. Also, individuals that do not wear underwear outside or after or during intercourse are prone to infections [7].

Primary treatment of myiasis includes mechanical extraction of maggots, antibiotics for coexisting infection, pain management, treatment of predisposing factors, and alleviation of risk factors [10]. The antiparasitic drug ivermectin has been successfully used in the treatment of myiasis [15]. In our case, we used turpentine oil for mechanical extraction and broad-spectrum antibiotics as the primary management.

A significant number of the adult population in the rural areas of developing countries like Bangladesh are illiterate and are not familiarized with education regarding hygiene. So, clinicians should emphasize the need for a careful genital exam to identify possibility of rare diseases. The doctor's role in educating patients is also important and must include the stimulation of the development of good hygienic habits among all patients. Simple acts such as regular soap-and-water bathing can prevent diseases such as the one we have described.

## 4 Conclusions

Parasitic infestation in humans is a rare phenomenon unless immunocompromised, a large portion takes place due to a lack of personal hygiene and sanitation. The utmost importance of public awareness for women in rural settings and the removal of social stigmata seeing such problems in women must be dealt with meticulously.

## Patient consent

Written informed consent was obtained from the patient for publication of this case report and accompanying images. A copy of the written consent is available for review by the Editor-in-Chief of this journal on request.

## Provenance and peer review

Not commissioned, externally peer-reviewed.

## Ethical approval

According to the institutional review committee publishing a case report does not require a letter of approval. So, ethical approval is not applicable to our case report.

## Funding

None.

## Guarantor

First author.

## Research registration number

Not applicable.

## CRediT authorship contribution statement

ABS: conceptualization; ABS, AM, SH: Patient care (diagnosis, treatment and follow-up); SH, AM: patient information collection; SS, MHN, PP, SH: manuscript writing and revision. All authors approved the final version.

## Declaration of competing interest

None.
